# Genome-Wide Investigation and Co-Expression Network Analysis of SBT Family Gene in *Gossypium*

**DOI:** 10.3390/ijms24065760

**Published:** 2023-03-17

**Authors:** Tianxi Xue, Lisen Liu, Xinyi Zhang, Zhongqiu Li, Minghao Sheng, Xiaoyang Ge, Wenying Xu, Zhen Su

**Affiliations:** 1 State Key Laboratory of Plant Environmental Resilience, College of Biological Sciences, China Agricultural University, Beijing 100193, China; 2 State Key Laboratory of Cotton Biology, Institute of Cotton Research, Chinese Academy of Agricultural Sciences, Anyang 455000, China

**Keywords:** cotton, SBT, co-expression network, gene family, salt stress

## Abstract

Subtilases (SBTs), which belong to the serine peptidases, control plant development by regulating cell wall properties and the activity of extracellular signaling molecules, and affect all stages of the life cycle, such as seed development and germination, and responses to biotic and abiotic environments. In this study, 146 *Gossypium hirsutum*, 138 *Gossypium barbadense*, 89 *Gossypium arboreum* and 84 *Gossypium raimondii* SBTs were identified and divided into six subfamilies. Cotton SBTs are unevenly distributed on chromosomes. Synteny analysis showed that the members of SBT1 and SBT4 were expanded in cotton compared to *Arabidopsis thaliana*. Co-expression network analysis showed that six *Gossypium arboreum* SBT gene family members were in a network, among which five SBT1 genes and their *Gossypium hirsutum* and *Arabidopsis thaliana* direct homologues were down-regulated by salt treatment, indicating that the co-expression network might share conserved functions. Through co-expression network and annotation analysis, these SBTs may be involved in the biological processes of auxin transport, ABA signal transduction, cell wall repair and root tissue development. In summary, this study provides valuable information for the study of SBT genes in cotton and excavates SBT genes in response to salt stress, which provides ideas for cotton breeding for salinity resistance.

## 1. Introduction

The precise regulation of protein levels ensures the normal function of plant cells, and the regulation of protein levels depends on the balance of protein synthesis and degradation. Since proteolysis is inherently irreversible, proteases control many aspects of plant growth, development and defense by selectively degrading regulatory proteins [1]. Most proteases in plants are of the catalytic serine peptidase type. Among serine peptidases, those related to bacterial subtilisin constitute the largest family, so it is very important to study the role of the SBT gene family in plant growth and development regulation and the response to environmental regulation [2,3].

Subtilisin-like serine protease (SBT) is a family of serine proteolytic enzymes that hydrolyze proteins into small peptides, which bind to receptors as signal molecules or ligands and participate in signal transduction. It has been reported in many plants for its involvement in various cellular processes such as protein activation. This unique region is used as a binding site for specific substrates [3]. There is a highly conserved catalytic triplet in the S8 peptidase domain, and amino acid residues consisting of aspartic acid (Asp), histidine (His) and serine (Ser) are characteristic of the subtilase family [4]. In addition, certain subtilase enzymes may also have a conserved catalytic asparagine (Asn) residue in the same S8 peptidase domain [4,5]. In addition, the protease-related (PA) domain, the C-terminal fibronectin (Fn)-III-like domain and the inhibitory (I9) domain have also been found in plant SBT gene family members [2], and the PA domain is often described as a protein–protein interaction module [6]. The PA domain interacts with substrates and contributes to substrate selectivity in plant SBT [7]. The C-terminal fibronectin (Fn)-III-like domain is a more general feature of plant SBT, functioning in certain plants [8], but not all SBT have this domain. As to SlSBT3, the C-terminal was inserted into a hydrophobic pocket near the substrate binding channel, thus stabilizing the loop system near the active site [2,8]. Inhibitor I9 has two functions. First, it assists in folding and thus requires the enzyme to mature during its passage through the secretory pathway. Inhibitor I9 deletion mutants do not pass endoplasmic reticulum (ER) quality control and remain in cells [8]. Second, Inhibitor I9 inhibits the activity of self-acting proteases. The conserved domain of SBTs is closely related to its multifunctional function in plants [8].

The SBT gene exists throughout the plant kingdom and plays a variety of roles in plant growth and defense [2]. The SBT family has been extensively studied in *Arabidopsis thaliana*. A total of 56 SBT family members have been identified [9]. AtSBT1.2 is specifically expressed in stomatal precursor cells and has the function of regulating stomatal density [10,11]. AtSBT1.4 plays an important role in regulating ABA signaling and drought tolerance through its interaction with OST1 [12]. Subtilase, which promotes the formation of the signal peptide AtSBT2.4, was found to be involved in embryonic development. Transposon markers identified AtSBT2.4 as the gene responsible for impaired epidermal function and conditional pathogenicity in *ale1* mutants [13,14]. Overexpression of AtSBT5.4 is used as a means of densifying organs produced by plants, inflorescences or floral meristem [15]. This trait may have important implications for biomass, fruit or flower production in other plants. In addition, AIR3 (AtSBT5.3) promotes lateral root formation [16], ARA12 (AtSBT1.7) participates in seed coat development [17] and XSP1 (AtSBT4.14) regulates xylem differentiation [18]. They not only play a role in plant-specific developmental processes, but also participate in the response of *Arabidopsis thaliana* to environmental stress. AtSBT3.5 was shown to participate in PME processing during root development [19]. AtSBT6.1 encodes a protein required for the activity of bZIP17 in the endoplasmic reticulum stress signaling pathway and has been observed in plants in response to salt stress [20,21,22,23]. In addition, the loss of AtSBT3.3 function in the deletion mutant impaired innate immunity, while the overexpression of AtSBT3.3 enhanced plant resistance to pathogens [24]. In cotton SBTs, GbSBT1 protein interacted with GbVE1, a verticillium-wilt-resistant protein, and GbSBT1 gene was up-regulated in response to verticillium wilt, jasmonic acid and ethylene. GbSBT1 silence increased the sensitivity of cotton plants to *Verticillium dahliae* infection [25].

Cotton is the main source of natural fiber for textile industries around the world. The genus *Gossypium* consists of 5 tetraploid (2n = 4 × 13 = 52) and 45 diploid (2n = 2 × 13 = 26) species, which are presumed to have originated from the common ancestor 5~10 MYA. *Gossypium hirsutum* (2n = 4 × 13 = 52) is expected to undergo an allopolyploid event at about 1~2 MYA, involving A and D genome progenitors [26]. The ancestor of *Gossypium arboreum* (AA, 2n = 2 × 13 = 26) may have been the donor of A subgenomes, while the ancestor of *Gossypium raimondii* (DD, 2n = 2 × 13 = 26) may have evolved into A allotetraploid cotton species by adding D subgenomes to *Gossypium hirsutum* (AADD, 2n = 4 × 13 = 52). Polyploid not only changes genome organization and gene expression, but also leads to the generation of multiple duplicate genes in the genome [27,28,29,30]. The publication of the genomic data of *Gossypium raimondii*, *Gossypium arboreum*, *Gossypium hirsutum* and *Gossypium barbadense* provides an opportunity for comparative analysis and the identification of key gene families among cotton species. Cotton is a model crop for studying polyploid evolution, as with the putative cotton and allotetraploid cotton genome decoded, scientists can learn about the molecular mechanism of cotton genome size variation, the formation and evolution of allopolyploid, the development and utilization of genetic resources and important agronomic traits, the basis for the breeding of new varieties, and to provide guidance for cotton. At the same time, with the continuous development of transcriptome sequencing technology, more and more transcriptome data are available at different stages of cotton development and under different environmental conditions. As genomics and transcriptomics data continue to grow, scientists are integrating them to build cotton databases such as CottonGen [31] and ccNET [32]. The CottonGen database provides reference genomes for different cotton species and the ccNET database can query the co-expression network of *Gossypium arboreum* and *Gossypium hirsutum* and infer the molecular functions and biological processes involved in cotton genes. These databases provide a basis for joint multi-omics analysis to explore candidate genes for key agronomic traits.

In general, drought and salt affect all levels of cotton, from the molecular to the biological levels, which leads to a decrease in plant growth, villus production and fiber quality. This effect varies with the timing and intensity of salt or drought stress, the growth stage and species. Drought has a negative effect on all cotton growth stages. However, seedlings, flowering and boll developmental stages are the most sensitive to water scarcity. Although several studies have been conducted to improve abiotic stress tolerance in cotton, breeding achievements have fallen short of expectations due to limited tolerance germplasm resources and poor understanding of the genetic basis of complex traits such as abiotic stress tolerance. Signal transduction plays a crucial role in plant responses to different stress conditions. Most SBTs are involved in signal transduction pathways. Plant SBTs affect all stages of the life cycle as they contribute to embryogenesis, seed development and germination, cuticle formation and epidermal patterning, vascular development, programmed cell death, organ abscission, senescence, and plant responses to their biotic and abiotic environments. We wish to identify some candidate SBT genes for cotton molecular breeding in resistance to abiotic stresses. At present, the function of the SBT gene in *Gossypium hirsutum*, *Gossypium arboreum*, *Gossypium raimondii* and *Gossypium barbadense* is rarely reported. Therefore, more detailed and comprehensive studies on the molecular evolution and gene function of the cotton SBT family are needed.

In this study, we first identified the SBT gene family members of *Gossypium arboreum* (GaSBT), *Gossypium raimondii* (GrSBT), *Gossypium hirsutum* (GhSBT) and *Gossypium barbadense* (GbSBT) and classified these SBT gene family members through phylogenetic analysis of *Arabidopsis thaliana* and four cotton species. Next, we used collinearity analysis to understand the underlying genetic mechanism of SBT gene family evolution. Then, the function of SBT gene family members in cotton was predicted by a co-expression network query. This study provides a reference for the structure and function of the SBT gene in cotton, and identifies genes related to salt stress by co-expression network analysis and the prediction of the biological processes involved by its members, which provides a research basis for the molecular breeding of cotton.

## 2. Results

### 2.1. Identification of SBT Gene Family Members in Cotton

To better explore the character of the SBT gene family in cotton, we selected two allotetraploid cottons, *Gossypium hirsutum* and *Gossypium barbadense*, and their possible ancestral diploid group A donor *Gossypium arboreum* (A2 genome) and diploid group D donor *Gossypium raimondii* (D5 genome).

We applied multiple methods including keyword query, domain query and BLAST search, and identified 146 *Gossypium hirsutum*, 138 *Gossypium barbadense*, 89 *Gossypium arboreum* and 84 (130 isoforms) *Gossypium raimondii* SBT gene family members (Table 1). There were 69 SBT genes in *Gossypium hirsutum* A subgenome, 74 in D subgenome and 3 in scaffold, and there were 67 members in *Gossypium barbadense* A subgenome, 70 in D subgenome and 1 in scaffold, thus showing there are more SBT gene family members in the D subgenome than in the A subgenome in allotetraploid cotton. Compared with ancestral diploids and heterotetraploids, the number of subgenomic SBT gene family members in *Gossypium hirsutum* and *Gossypium barbadense* was less than that in *Gossypium arboreum* and *Gossypium raimondii*, indicating that there might be gene loss in SBT gene family members in cotton during the evolutionary process.

The SBT gene family has been identified in many species, including *Arabidopsis thaliana* (56 gene members) [9], rice (Oryza sativa, 63) [33], poplar (*Populus trichocarpa*, 90) [3], potato (Solanum tuberosum, 82) [34], pineapple (*Ananas comosus* L, 54) [35] and grape (*Vitis vinifera*, 82) [36,37]. Compared to these plants, the number of SBT gene family members is higher in cotton species. As to functional domains in SBT proteins, similar to other species, SBT gene family members had at least one key domain (Inhibitor I9, PA, Peptidase S8, FnIII-like domain, TPPII) (Table S1), indicating that the functional diversity of cotton SBTs is related to its multiple protein domains. The protein lengths of SBT gene family members were different in allotetraploid cotton. *Gossypium hirsutum* was 54 (Gh_Sca122440G01) to 1393 (Gh_A08G0882) aa and *Gossypium barbadense* was 89 (GB_scaffold13001_objG0001) to 3521 (GB_D10G2624) aa (Table S1). We found that *Gossypium barbadense* has a wider range of protein lengths than *Gossypium hirsutum*. However, the differences in protein length were small in the ancestral diploids of cotton. *Gossypium raimondii* was 77 (Gorai.011G157100.1) to 1351 (Gorai.004G002500.1) aa, and that of *Gossypium arboreum* was 84 (Cotton_A_30414) to 1415 (Cotton_A_35466) aa. In *Arabidopsis thaliana*, the protein sizes of 56 SBT members ranged from 671 to 1396 aa [9]. Compared with *Arabidopsis thaliana*, cotton has a wider range of amino acid lengths.

### 2.2. Phylogenetic Analysis of SBT Gene Family Members in Cotton

**Figure 1:**
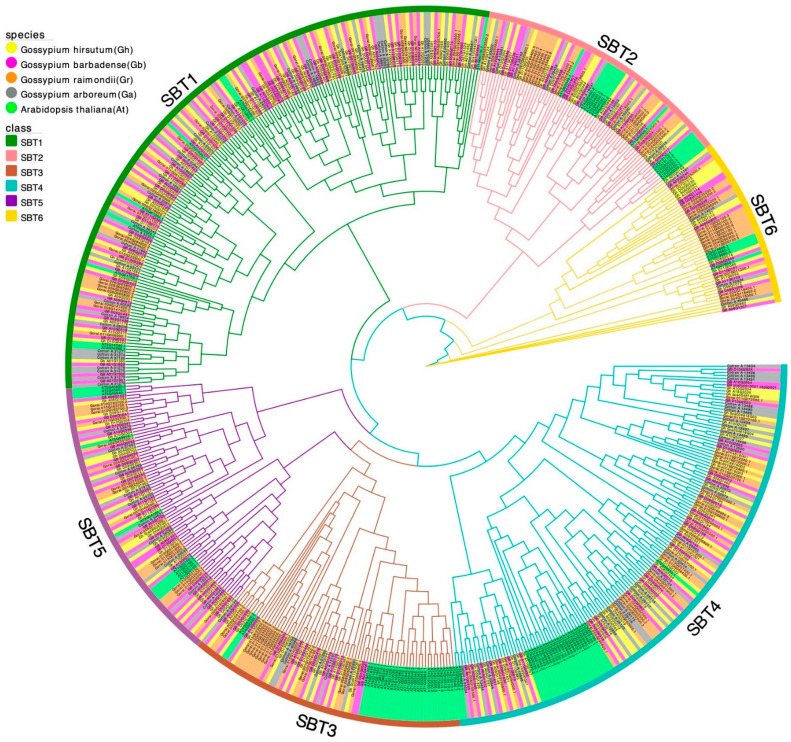
Phylogenetic relationships of SBT in different species. The circles around the phylogenetic tree (from center to periphery) represent each SBT subfamily and the distribution of species in different colors. Dark green, pink, brown, cyan, purple and dark yellow lines represent subfamilies from SBT1 to SBT6, respectively. Different gene names and background colors indicate different species. Yellow, dark pink, orange, grey and green indicate *Gossypium hirsutum, Gossypium barbadense, Gossypium raimondii*, *Gossypium arboretum* and *Arabidopsis thaliana*, respectively.

In order to determine the taxonomic and evolutionary relationships of SBT gene family members in cotton, we used a phylogenetic tree, multiple sequence alignment, BLAST search, domain analysis and manual correction to classify SBT gene family members. In addition, *Arabidopsis thaliana* was used as the classification standard for cotton SBT because it is a model species. We took the sequences of 91 *Arabidopsis thaliana* SBT proteins (56 genes), 146 *Gossypium hirsutum* SBT proteins, 138 *Gossypium barbadense* SBT proteins, 130 *Gossypium raimondii* SBT proteins (84 genes) and 89 *Gossypium arboreum* SBT proteins (Table S1) and constructed a maximum likelihood phylogenetic tree (Figure 1). The SBT gene family was divided into six subfamilies (SBT1, SBT2, SBT3, SBT4, SBT5 and SBT6), which is consistent with the previously reported classification of SBT families in *Arabidopsis thaliana* [9], *Ananas comosus* L [35] and *Vitis vinifera* [36,37]. These results confirm the high degree of the evolutionary conserved nature of the SBT family in plants. In each subfamily, we found that SBT of the four cotton species tended to cluster together, and the ancestral diploids and heterotetraploids all appeared in the same branch, indicating that the four cotton species were closely related and evolutionarily conserved.

As shown in Table 1, the number and distribution of SBT subfamilies of the four cotton species were similar. Among all SBT gene members, SBT1 gene family members were the most, and SBT6 gene family members were the least. Since SBT1 and SBT4 were more abundant in the four cotton species than in the other subfamilies, we hypothesized that the reason for the high number might be caused by gene duplication and expansion during the evolutionary process, so we studied the formation of the SBT gene family by collinearity analysis. Past studies have found that *Arabidopsis thaliana* SBT1 has nine gene family members and SBT6 has six [9]. Compared to *Arabidopsis thaliana*, the much greater number of SBTs in cotton indicates that the SBT1 members in cotton might have a broader function. However, cotton SBT6 is the smallest subfamily, which is consistent with the results of *Arabidopsis thaliana,* suggesting that this subfamily may not have undergone evolutionary differentiation between dicotyledons and monocotyledons. In addition, compared with *Arabidopsis thaliana* in the SBT4 and SBT5 subfamilies’ classification, some *Arabidopsis thaliana* SBT gene family members are in a branch, while the other subfamilies do not have the above results (Figure 1). In the SBT4 and SBT5 subfamilies, *Arabidopsis thaliana* and cotton may have great sequence differences.

We selected one protein each for the six subfamilies of *Arabidopsis thaliana* and *Gossypium arboreum* and obtained their predicted three-dimensional protein structures by using alphafold2. In previous studies, SBT has the Peptidases S8 domain, which has three enzyme active sites [2]. We compared the reported three-dimensional structure of the SBT protein sequences with the SBT proteins of the six subfamilies of *Arabidopsis thaliana* and *Gossypium arboreum* to find the locations and domains of Asp, His and Ser residues in catalytic triplets in the predicted three-dimensional structure of proteins (Figures S1 and S2).

The predicted PA, FnIII, Peptidases S8 domains and catalytic triplet structures are similar to those reported for the protein three-dimensional structures of cucumber and SISBT3 [2], suggesting that these domains are structurally conserved in *Arabidopsis thaliana* and *Gossypium arboreum*. A comparison between the structures of each subfamily found that only the Peptidases S8 domain of SBT6 differed the most from the other subfamilies, indicating differences in function. The different positions of the Inhibitor I9 domain in different subclasses, such as SBT1 and SBT3 on the left of the Peptidases S8 domain and SBT2, SBT4 and SBT5 behind Peptidases S8, suggest that each subfamily may be different in inhibiting its own enzyme activity through the Inhibitor I9 domain. In addition, random curling around the β-sheet of the FnIII domain in each subclass was found to be different, and FnIII had a hydrophobic effect, further improving the stability of the enzyme (Figures S1 and S2).

### 2.3. Gene Structure Analysis of SBT Gene Family Members in Cotton

Divergences in coding regions, especially those that may change the function of the gene, can be caused by amino-acid-altering substitutions and/or alterations in the exon–intron structure. To further understand their gene structure diversity, we analyzed the exon–intron organization of cotton SBT genes.

**Figure 2:**
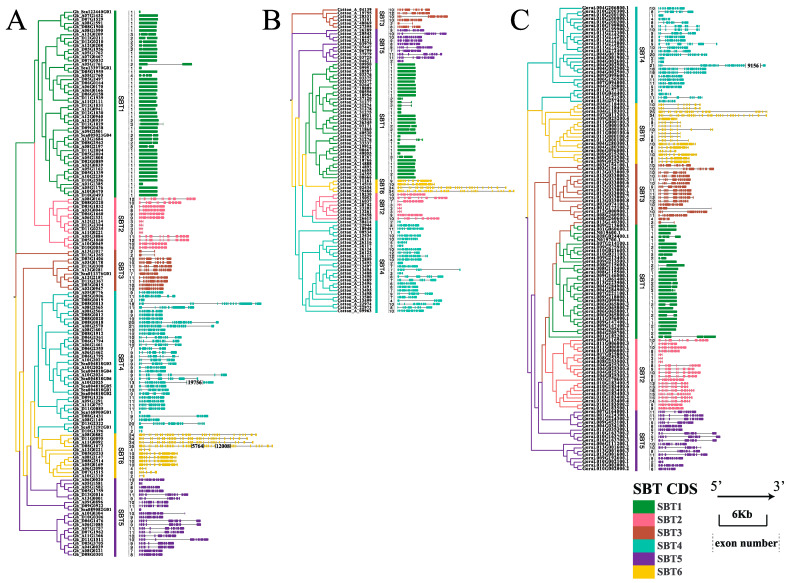
SBT gene structure of cotton. (**A**) SBT gene structure of *Gossypium hirsutum*. The phylogenetic tree is built by FastTree. (**B**) Genetic structure of *Gossypium arboretum*. The phylogenetic tree is built by FastTree. (**C**) Isoform structure of *Gossypium raimondii*. Different gene models as a result of alternative splicing or alternative start codons are numbered consecutively as Gorai.0XXGXXXXXX.1, .2, etc. The evolution tree is built by FastTree. The squares are CDS, the horizontal lines are introns. Dark green, pink, brown, cyan, purple and dark yellow lines represent subfamilies from SBT1 to SBT6, respectively. The dotted line is exon number.

The conserved gene structure of each subgroup suggests that SBT genes with high homologous sequence similarity tend to have the similar gene structure. There was little difference in the number of exons of the SBT subfamily among the four cotton species, indicating that SBT was evolutionarily conserved in the four cotton species. On the other hand, there were significant differences in the number of exons among the six SBT subfamilies among the four cotton varieties. The average number of exons in SBT6, SBT2– SBT5 (SBT2, SBT3, SBT4 and SBT5) and SBT1 was 14, 9 and 1, respectively. We found a significant difference in the number of exons between SBT1 and SBT6 (Figure 2 and Figure S3), which suggests that SBT1 and SBT6 gene structures diverge to improve the fitness of the organisms [38]. These results are consistent with those of *Arabidopsis thaliana* (SBT1: average exon number 1, SBT6: average exon number 22).

To further understand the gene structure of SBT1 and determine whether it is conserved in other species. The number and proportion of SBT1 genes without introns were counted in four cotton species, and were 74% in *Gossypium hirsutum* (37/50), 70.59% in *Gossypium barbadense* (36/51), 55.5% in *Gossypium raimondii* (15/27) and 76.47% in *Gossypium arboreum* (26/34) (Figure 2 and Figure S3). These results suggest that most SBT1 gene family members of the four cotton species did not have introns, which was consistent with the structural characteristics of the SBT gene in *Arabidopsis thaliana* (7/9, 77.78%) [9] and *Ananas comosus* L (12/30, 40%) [35]. In addition to the gene structure, we also analyzed the domain of SBT1 and found that most proteins have four key domains (Inhibitor I9, Peptidases S8, PA domains and FnIII-like domain) (Figures S1, S2 and S4).

In SBT1 branches of the evolutionary tree, homologous genes share the same domain, gene length and exon number. For example, the SBT1.8 tree branches into two groups. AtSBT1.8 in a separate clade indicates that it evolved in parallel with cotton SBTs. Cotton_A_33337 in SBT1.8 is in a branch with the D genome and is included in the CottonGen database [31], with only one domain. Except for Cotton_A_33337, the subgenomes of the remaining genes were in a branch with the corresponding ancestral diploid genome, and the heterotetraploid had twice as many memories as the ancestral diploid. Compared with other SBT1 clades, SBT1.8 was mainly composed of two exons in the gene structure analysis. At the same time, SBT1.8 contained two domains in the domain analysis. The first domain was all four key domains (Inhibitor I9, Peptidases S8, PA domain and FnIII-like domain). The second was multiple AprE based on the four key domains (Figure S4).

In addition, three Inhibitor I9, Peptidases S8, PA domains and FnIII-like domains were found in GB_A10G2665 and GB_D06G2013. GB_A10G2664 has five Inhibitor I9, Peptidases S8, PA domains and FnIII-like domains, and GB_D10G2624 has four Inhibitor I9, Peptidases S8 and PA domains, indicating that domain replication events may occur during the evolution of this gene (Figures S3 and S5 and Table S1). These genes belong to GbSBT4, and we suspect that *Gossypium barbadense* SBT4 has a gene expansion phenomenon.

### 2.4. Chromosomal Localization, Collinearity Analysis and Selective Pressure Analysis of the SBT Family Genes in Cotton

**Figure 3:**
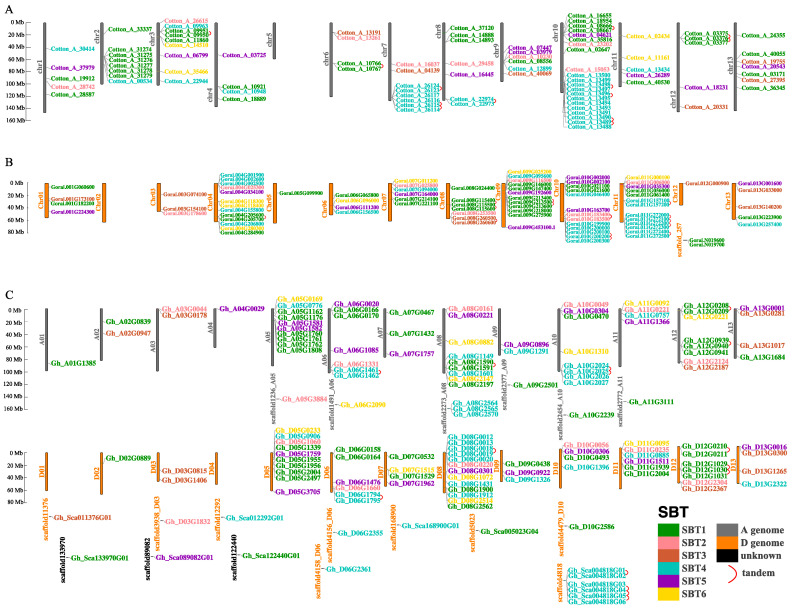
Chromosome positions of SBT gene family members in cotton. (**A**) *Gossypium arboreum*. (**B**) *Gossypium Raimondii.* (**C**) *Gossypium hirsutum*. The grey bars represent the chromosomes of the A subgenome. The orange bars show the chromosomes of the *Gossypium Raimondii* D subgenome. Black represents unpredicted subgenomic classifications. Different color gene names represent different classifications. Dark green, pink, brown, cyan, purple and dark yellow lines represent subfamilies from SBT1 to SBT6, respectively. The red curve connects tandem repeats, gene locations and chromosome sizes estimated in Mb on the left side of the figure.

To better understand the distribution of SBT on the chromosome, genome and subgenome of cotton, a chromosome map of SBT was constructed based on the genome sequences of the four cotton species. We found that SBT is unevenly distributed on the chromosomes in the four cotton species (Figure 3 and Figure S6). In the case of *Gossypium hirsutum*, first of all, the four cotton species differ in the number of genes on each chromosome. In all chromosomes, D08 chromosome has the most SBT genes, but D01 and D04 have no SBT gene. Second, the distribution of SBT genes on a single chromosome is not uniform, and some SBT genes exist in gene clusters, most of which are SBT1 and SBT4.

Gene families commonly arise because of gene duplication events, mainly including tandem, segmental and whole-genome duplications. We used synteny analysis to understand how the SBT gene family was formed. We found that there were larger fragment duplications than tandem duplications in the four cotton species, indicating that the SBT gene family was formed mainly through segmental duplication. In tandem duplication, SBT1 and SBT4 accounted for more than any other subfamily (Figure 3 and Table S2). This result may indicate that most of the gene clusters on a single chromosome are SBT1 and SBT4. At the same time, collinearity analysis was performed on selected portions of tandem duplication genes of SBT1 and SBT4. In SBT1 tandem genes, the collinear genes corresponding to Cotton_A_03375, Cotton_A_03376 and Cotton_A_03377 are Gh_A12G0939, Gh_A12G0940 and Gh_A12G0941 on chromosome A12, meanwhile, they are Gh_D12G1029, Gh_D12G1030 and Gh_D12G1031 on chromosome D12. In SBT4 tandem genes, the collinear genes corresponding to Cotton_A_13490, Cotton_A_13495, Cotton_A_13497 and Cotton_A_13498 are Gh_A10G2024, Gh_A10G2025, Gh_A10G2026 and Gh_A10G2027 located on chromosome A10, as well as Gh_Sca004818G06, Gh_Sca004818G05, Gh_Sca004818G04 and Gh_Sca004818G03 located on chromosome scaffold4818. These results suggest that the selected tandem duplication genes of SBT1 and SBT4 in cotton are conserved in the evolution of tetraploid cotton and in diploid cotton (Figure S7).

In addition, the nonsynonymous (Ka) to synonymous (Ks) substitution rate ratio (Ka/Ks) was used to serve an estimator for selective pressure on DNA sequence evolution. Selective pressure analysis showed that most of the Ka/Ks pairs of homologous genes were less than 1 (Table S2), indicating that the cotton SBT gene was a purified selection.

### 2.5. Co-expression Network Analysis of Gossypium arboreum SBT

Compared with other cotton species, *Gossypium arboreum* has advantages in terms of its stress resistance [39]. In order to explore the function of the SBT gene in cotton, we used the ccNET [32] co-expression network database to analyze the expression pattern of the *Gossypium arboreum* SBT gene in root, stem and leaf tissues and under drought and salt stresses.

**Figure 4:**
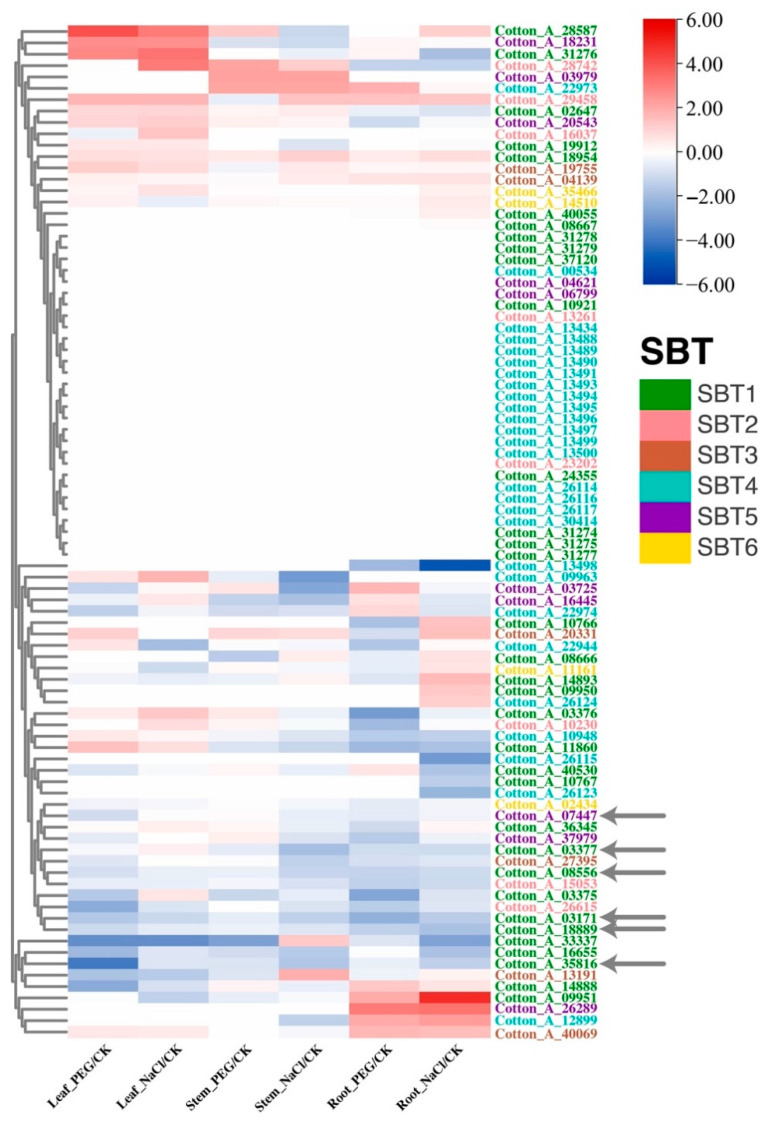
Expression profiles of 89 SBT gene families in *Gossypium arboreum*. There are 89 *Gossypium arboreum* gene family members in the vertical, and the ratio of PEG and NaCl treatment to control in leaf, stem and root tissues in the horizontal sequence, and the classification of each SBT gene family member is at the far right. Different color fonts indicate the subfamilies from SBT1 to SBT6. Dark green, pink, brown, cyan, purple and dark yellow lines represent subfamilies from SBT1 to SBT6, respectively.

The *Gossypium arboreum* and *Gossypium hirsutum* SBT gene family members were divided into three groups by expression profile clustering analysis. The first type is the up-regulation of most genes under salt and drought stresses, the second type is the unchanged expression and the third type is the down-regulation of most genes under salt and drought stresses. In the first category, every subfamily is present and evenly distributed, in the second category, most are SBT4, and in the third category, every subfamily is present but the number of SBT1 is the highest (Figure 4 and Table S3). In addition, we found that there was little difference in the expression trend between the A subgenome and the D subgenome of *Gossypium hirsutum*. The gene expression trend of *Gossypium hirsutum* gene family members in salt treatment and PEG treatment was the same as that in *Gossypium arboreum*, which further indicated that diploid cotton and allotetraploid cotton were conserved (Figure S8 and Table S4).

**Figure 5:**
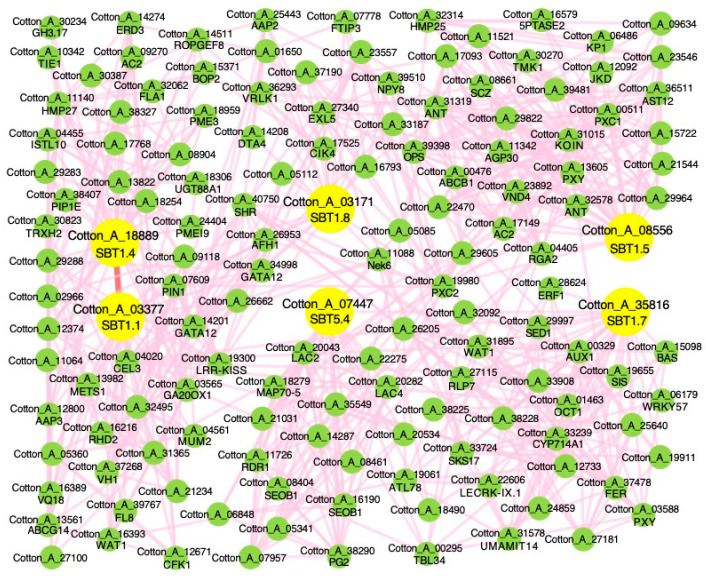
Gene co-expression network of six *Gossypium arboreum* SBT gene family members. The green nodes are co-expressed genes, and the large yellow nodes are *Gossypium arboreum* SBT genes. The pink line shows a co-expression relationship between two genes and the red line indicates SBT gene co-expression.

As shown in Figure 4, genes with a similar variation trend of gene expression were gathered together, and we conducted a co-expression network query for the genes clustered in the same category in the ccNET database [32]. We found that six *Gossypium arboreum* SBT gene family members (Cotton_A_03171, Cotton_A_03377, Cotton_A_35816, Cotton_A_08556, Cotton_A_18889, Cotton_A_07447) were found in one network that consists of 139 co-expressed genes (Figure 4 and Figure 5, Table S3). To explore the biological function of this network, we used AgriGOv2 [40] to screen the functional modules in the co-expression network and annotated the genes through a literature search. It was found that these six SBT gene family members of *Gossypium arboreum* were enriched in serine-type endopeptidase activity. In the DAG enrichment diagram (Figure S9), kinase activity and protein phosphorylation terms were significantly enriched, and protein kinases catalyzed the phosphoric reaction of proteins, and receptor protein kinases were found in these terms (Table S5). These results indicate that the network is involved in signal transduction and affects cotton growth and development.

Because *Arabidopsis thaliana* is a dicotyledonous model plant, we searched for *Arabidopsis thaliana* orthologous genes co-expressed in *Gossypium arboreum* to further predict cotton gene function. We found many receptor kinases in gene sets with terms such as protein phosphorylation, transmembrane receptor protein tyrosine kinase signaling pathway, plasma membrane, ATP binding and plant-type cell wall (Figure S9 and Table S4). Among receptor kinases, AtTMK1 is related to root tissue development [41] and LRR-KISS responds to salt stress [42]. PXC1 may be involved in the regulation of cell elongation or swelling [43], and AtVH1 affects the proliferation and differentiation of many cells in young leaves [44]. Cheng Huang et al. found that down-regulation of AtVRLK1 could promote the thickening of the secondary cell wall, while up-regulation of AtVRLK1 could enhance cell elongation and inhibit the thickening of the secondary cell wall [45]. These results suggest that receptor kinases in the co-expression network of *Gossypium arboreum* may be involved in root development and the response to salt stress.

Therefore, we performed cluster analysis on the gene expression profiles of the six SBT co-expressed gene networks in different tissues under abiotic stresses and found that most genes were down-regulated in the roots, stems and leaves of *Gossypium arboreum* after salt treatment and drought treatment. Five of the six SBT gene family members (Cotton_A_03171, Cotton_A_03377, Cotton_A_35816, Cotton_A_08556, Cotton_A_18889) were significantly down-regulated (Figure S10A, Table S6). We found that receptor kinases PXC1 [43], VH1 [44], VRLK1 [45] and lecrk-IX.1 [46] were down-regulated, SCZ [47] and SHR [48] related to root tissue development (Table S4) were down-regulated under salt treatment, and AUX1 [49] and ABCB1 [49] related to polar auxin transport were down-regulated (Figure S10b), suggesting that SBT1 was involved in auxin transport, root tissue development and the response to salt stress.

### 2.6. Comparison of Co-Expression Networks between Arabidopsis thaliana and Different Cotton Species

In addition to the five SBT1 genes in *Gossypium arboreum* that were down-regulated by salt treatment in the root tissue, we further investigated the co-expression network of five direct SBT1 orthologous genes in *Gossypium hirsutum* and *Arabidopsis thaliana*, in order to compare the network conservative types between species.

**Figure 6:**
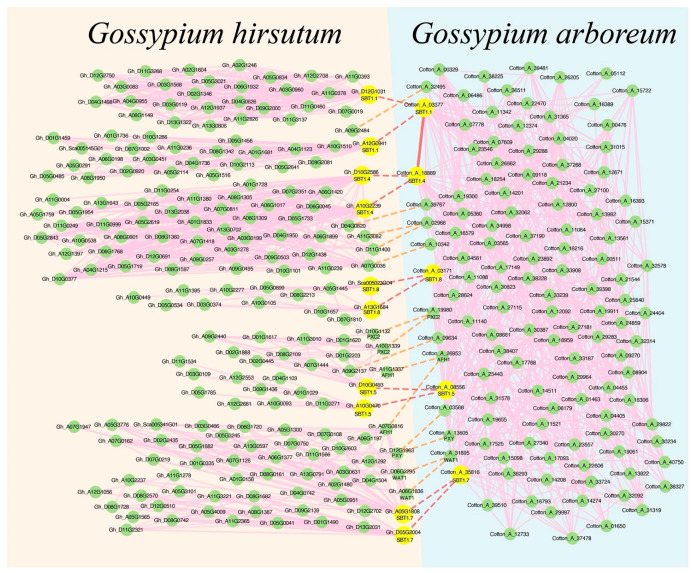
Comparison of SBT network between *Gossypium arboreum* and *Gossypium hirsutum*. The green nodes are co-expressed genes, and the large yellow nodes are *Gossypium arboreum* and *Gossypium hirsutum* SBT genes. *Gossypium arboreum* co-expressed network is blue background and *Gossypium hirsutum* is yellow. The pink line shows a co-expression relationship between two genes and the red line indicates SBT gene co-expression. The red dot line indicates that SBT is lineally homologous between two cotton species and the orange dot line indicates that co-expressed genes are lineally homologous between two cotton species.

Firstly, five SBT1 were analyzed by collinearity analysis and a ccNET database [32] query to obtain direct homologous genes of *Gossypium hirsutum* corresponding to *Gossypium arboreum* (Figure S11) and to query the SBT co-expression network of *Gossypium hirsutum*. We found that Gh_D10G0493, Gh_A13G1684 and Gh_Sca005023G04 corresponding to Cotton_A_08556 and Cotton_A_03171 had collinearity in *Gossypium hirsutum* (Table S7), and were in the same co-expression network (Figure 6 and Table S8). The co-expression network and annotation analysis showed that the leucine-rich repeat protein kinase family (PXY and PXC2) of *Gossypium arboreum* and its homologous *Gossypium hirsutum* genes were involved in signal transduction. According to the literature notes, multiple families of leucine-rich repeat protein kinases were found to be involved in root growth and cell wall formation (Table S5). These results indicate that the co-expression networks of both *Gossypium hirsutum* and *Gossypium arboreum* are involved in signal transduction pathways.

Then, to compare the conservation of *Arabidopsis thaliana* and the cotton co-expression networks, we queried the *Arabidopsis thaliana* co-expression networks in the ATTED-II database. The *Arabidopsis thaliana* orthologous genes corresponding to *Gossypium arboreum* SBT genes were obtained from the CottonGen database [31], and we found that AT2G05920, AT5G67360, AT3G14067, AT1G01900 and AT3G14240 are on the same network (Figure S12a and Table S9). Finally, SEACOMPARE analysis was performed on *Gossypium arboreum*, *Gossypium hirsutum* and *Arabidopsis thaliana* to explore the functional conserved networks. The co-expression network between the species involved in the biological processes of protein serine/threonine kinase activity, plant-type cell wall, transmembrane receptor protein tyrosine kinase signaling pathway, transmembrane transporter activity and serine-type endopeptidase activity were significantly enriched in *Arabidopsis thaliana*, *Gossypium arboreum* and *Gossypium hirsutum* (Figure S12b). SBT gene family members were enriched into serine-type endopeptidase activity. The contents of the transmembrane receptor protein Tyrosine kinase signaling pathway and protein phosphorylation were significantly enriched in *Arabidopsis thaliana*, *Gossypium arboreum* and *Gossypium hirsutum*. Protein kinases catalyze the phosphoric acid reaction of proteins and receptor protein kinases are found in these terms. Apoplast and the integral component of the membrane were significantly enriched in *Arabidopsis thaliana*, *Gossypium arboreum* and *Gossypium hirsutum* (Figure S12b). These results suggest that both the cotton and *Arabidopsis thaliana* co-expression networks are involved in signal transduction and affect plant growth.

As shown in Figure S12b, the response to water deprivation and the response to osmotic stress were enriched in *Gossypium hirsutum*. Therefore, expression profile clustering analysis was performed on transcriptome data related to the water stress of *Gossypium hirsutum* and *Arabidopsis thaliana* to determine the conserved type of network function.

The expression profile clustering analysis showed that most of the co-expressed genes in *Arabidopsis thaliana* and *Gossypium hirsutum* were down-regulated in salt treatment (Figures S10a and S12c,d, Tables S10 and S11). In addition, we found that five SBT1 genes in *Gossypium arboreum* and *Arabidopsis thaliana* were down-regulated by 6 h salt treatment in root tissues. Thus, we concluded that the co-expression network of *Gossypium arboreum* was conservative in *Gossypium hirsutum* and *Arabidopsis thaliana*.

## 3. Discussion

In this study, 146 *Gossypium hirsutum*, 138 *Gossypium barbadense*, 89 *Gossypium arboreum* and 84 *Gossypium raimondii* SBT gene family members were identified. There were 69 SBT genes in *Gossypium hirsutum* A subgenome, 74 in D subgenome and 3 in scaffold, as well as 67 members in the *Gossypium barbadense* A subgenome, 70 in the D subgenome and 1 in scaffold (Table 1). The SBT gene family was divided into six groups by BLAST and phylogenetic analysis. We found that most of the SBT1 gene family members in the four cotton species had no introns (Figure S4). Chromosome location and synteny analysis showed that SBT gene family members were not evenly distributed on each chromosome. The SBT gene family was mainly formed by segmental duplication, and SBT1 and SBT4 genes expanded significantly (Figure 3 and Figure S6). Combined with the results of gene structure analysis and collinearity analysis, it was found that cotton SBT1 expanded significantly, and the function of cotton SBT1 was mined and predicted. Co-expression network analysis revealed six SBT gene family members (Cotton_A_03171, Cotton_A_03377, Cotton_A_35816, Cotton_A_08556, Cotton_A_18889, Cotton_A_07447) in a network (Figure 4 and Figure 5). The annotation of the network revealed that the network was involved in signal transduction and root tissue growth and development. Five SBT1 (Cotton_A_03171, Cotton_A_03377, Cotton_A_35816, Cotton_A_08556, Cotton_A_18889) were down-regulated in response to salt stress in root tissue (Figure S10). We found five direct orthologous genes of SBT1 corresponding to *Gossypium hirsutum* (Figure S11) and *Arabidopsis thaliana* and queried the co-expression network. By enrichment analysis and expression profile clustering analysis, we found that the function of the network was conserved in *Gossypium arboreum*, *Gossypium hirsutum* and *Arabidopsis thaliana* (Figures S10 and S12).

### 3.1. Evolution of Cotton SBT Family Genes

In this study, we discussed the evolution of SBT family genes. Among the identified SBT members, we found that the number of allotetraploid subgenomes was less than the number of ancestral diploids. In allotetraploid cotton, there are more SBT gene family members in the D subgenome than in the A subgenome. These results indicate that there is asymmetry in gene loss among allotetraploid subgenomes, and the loss of the A subgenome is more common than that of the D subgenome. These are consistent with the study of Zhang et al. [39]. At the same time, the number of each subfamily was compared, and it was found that SBT1 and SBT4 lost more genes. Based on chromosomal location and collinearity analysis, it was found that there was an uneven distribution of the SBT gene on the A chromosome, and the SBT gene alignment of subgenome A to *Gossypium raimondii* and subgenome D to *Gossypium arboreum* showed that the subgenome had great structural variation, chromosomal cross swap and structural rearrangement after the formation of tetraploids. In conclusion, the SBT gene family analysis showed that the two subgenomes of tetraploid cotton showed asymmetric evolution in terms of the loss of genome structure rearrangement genes after the polyploidy event.

In addition, SBT gene structure analysis showed that there was little difference in the gene structure and exon number among the four cotton species in the same subfamily. In the protein domain analysis, all the SBT gene family members of the four cotton species had at least one key SBT domain. In transcriptional change analysis, there was little difference between the expression trends of subgenome A and subgenome D (Figure S8). SBT1 has the least number of exons among the six subfamilies. The simpler the gene structure means the easier it is for gene amplification; thus, SBT1 has the most gene family members. Among the five down-regulated SBT1 genes found in the co-expression network, SBT1.8 has two exons and very short introns on average, which may be the structure formed after gene expansion (Figures S4 and S10).

### 3.2. Cotton SBT Gene Function during Environmental Response

In order to understand the function of cotton SBT under salt stress, we analyzed the *Gossypium arboreum* co-expression network of SBT gene family members and found that six SBT were in the same network, among which five SBT1 genes were down-regulated in response to salt stress (Figure S10b). GO enrichment analysis and literature annotation analysis of the network showed that the network was involved in root tissue development in response to salt stress (Figure S9 and Table S5).

We identified genes associated with phytohormone synthesis in the *Gossypium arboreum* co-expression network. These genes play key roles in salt stress adaptation. We found that auxin-transport-related genes WAT1 [50], PIN1 [51], AUX1 [49] and ABCB1 [49] and root-tissue-development-related genes SHR [48], SCZ [47], VH1 [44], TMK1 [41] and CEL3 [52] were down-regulated in root tissue salt treatment. These results suggested that *Gossypium arboreum* SBT may be involved in auxin transport and root tissue development. Moreover, it was further demonstrated that auxin levels and the expression of auxin transporters were decreased under salt stress, which resulted in decreased root meristem activity and growth inhibition of primary roots [53]. Therefore, understanding the mechanisms regulating auxin signaling under salt stress will be useful for future biotechnology applications to improve plant salt tolerance [54]. In addition to auxin, the ABA signal transduction pathway is a research hotspot in a plant’s response to salt stress. *Gossypium arboreum* SBT1.4 was co-expressed with SBT1.1. The AtSBT1.4 mutant SASP showed enhanced ABA sensitivity during seed germination and seedling growth, which enhanced ABA-mediated leaf senescence [12]. SCR can directly regulate the expression of several transcription factors controlling ABA response [47]. These results suggest that both SBT1.1 and SBT1.4 in *Gossypium arboreum* may be involved in the ABA signal transduction pathway in response to salt stress affecting root growth and development. In the co-expression network of *Arabidopsis thaliana*, *Gossypium hirsutum* and *Gossypium arboreum*, many co-expressed genes were found to be receptor kinases (Figure S12b, Tables S5, S8 and S9). According to the literature query, the receptor kinase FER binds to RALF peptide, the homologous receptor of TDIF peptide is PXY and the small signal peptide formed by SBT hydrolysis may be RALF or TDIF [55]. In addition, we found AT3G13510 and AQI peptides in the *Arabidopsis thaliana* co-expression network, and these peptides may be obtained by SBT hydrolysis (Table S9).

SEACOMPARE analysis showed that *Gossypium arboreum*, *Gossypium hirsutum* and *Arabidopsis thaliana* were enriched to the cell wall (Figure S12b). In plants, cell walls not only determine cell expansion and growth but also provide mechanical protection against abiotic stress [56]. Accumulated evidence suggests that maintaining cell wall integrity is essential for plant salt tolerance and root tissue development. Most SBTs are targeted to the cell wall, where they contribute to the control of growth and development by regulating the properties of the cell wall and the activity of extracellular signaling molecules. AtSBT1.7 triggers the accumulation and/or activation of cell-wall-modifying enzymes that are required for external primary cell wall loosening or promoting mucus expansion, as indicated by increased pectin methyl esterase activity during AtSBT1.7 mutant seed development. FERONIA (FER) is a local receptor kinase in the plasma membrane, which is considered to be one of the cell wall integrity sensors. FER is involved in the general stress response by interacting with the abscisic acid (ABA) signaling pathway. The mutation of the FER gene leads to enhanced leaf bleaching and root cell swelling under salt stress [55]. It is proposed that FER is involved in the regulation of cell wall repair under salt stress through Ca^2+^-mediated signaling pathway. In the network, FER expression in *Gossypium arboreum* was down-regulated by salt treatment in root tissues (Figure S10b), suggesting that SBT may participate in the Ca^2+^-mediated signaling pathway in response to salt stress and participate in regulating cell wall biological processes [57]. In conclusion, SBT may be involved in the biological processes of auxin transport, ABA signal transduction, cell wall repair and root tissue development. We mined *Gossypium arboreum* SBT1 genes and their corresponding *Gossypium hirsutum* genes, and they will be verified in the future. Since cotton is not a food crop but a cash crop, it is important to mine the SBT gene associated with salinity tolerance to allow more fertile land to be used for food crops.

There are deficiencies in this study: First, at present, the co-expression network of *Gossypium barbadense* and *Gossypium raimondii* has not been constructed. More omics data should be provided to ensure the accuracy of the network and improve the prediction accuracy, so as to provide a reference for the subsequent mining of key agronomic trait genes and provide a basis for further comparison between diploid ancestors and heterotetraploids. Second, SBT genes related to root tissue development in response to salt stress need to be further verified by experiments. For example, compared with the four SBT1 down-regulated by other salt treatments, most SBT1.8 had two exons (Figure S4), and the gene down-regulated by salt treatment was obvious in the expression profile analysis (Figure 4 and Figure S8, Tables S3 and S4). With multiple omics data, we are able to study SBT to integrate genomics and transcription data analysis, and by combining the apparent omics three genomics data analysis, through the analysis of more restructuring, we could find cotton SBT under different stress levels and states of transcriptional expression and the contact between them, and further exploring more genes related to agronomic traits.

## 4. Materials and Methods

### 4.1. Identification of SBT Gene Family Members in Cotton

Genome sequence data for *Gossypium* ssp. were obtained from CottonGen database [31] (https://www.cottongen.org/, accessed on 15 September 2021) (*Gossypium hirsutum:* NAU-NBI_v1.1_a1.1*, Gossypium barbadense:* ZJU_v1.1_a1*, Gossypium arboreum:* CGP-BGI_v2_a1 and *Gossypium raimondii:* JGI_v2_a2.1). We identified SBT gene family members in the following three ways. First, we ran a genetic search on CottonGen (https://www.cottongen.org/, accessed on 30 September 2021) by typing “Subtilisin” into the search box. Second, downloaded Peptidase_S8 model from PFAM website and conducted HMMER search through HMMER software. Third, the *Arabidopsis thaliana* SBT protein sequences confirmed in the literature were aligned with cotton protein sequences by BLASTP, where the parameter of the e-value was less than 5. Gene family members were obtained by combining the above three methods. Molecular weight (MW) and theoretical isoelectric point (pI) were investigated with the TBtools [58]. Subcellular locations were predicted by online tools CELLO [59] (http://cello.life.nctu.edu.tw/, accessed on 3 March 2022). The domain of SBT gene family member was confirmed by NCBI-CDD [60] (http://www.ncbi.nlm.nih.gov/Structure/cdd/cdd.shtml, accessed on 3 March 2022), and the e-value was less than 1.

### 4.2. Sequence Alignment and Phylogenetic Analysis

The SBT gene family members of four cotton species and *Arabidopsis thaliana* were constructed using Muscle 5.1. Osx64 (muscle -super5 input.fa -output aln.afa) for multi-sequence comparison of only domain proteins, and the tree was constructed with the maximum likelihood method (model: VT+I+I+R4, predicted by Modelfinder) IQtree2.2 using domain protein sequence muscle comparison [61,62].

Phylogenetic tree of gene structure was built by FastTree [63]. *Gossypium hirsutum* and *Gossypium arboreum* used WAG model and *Gossypium barbadense* and *Gossypium raimondii* used JTT model.

### 4.3. Cotton Gene Structure, Location Display, Cotton Collinearity Analysis, Calculation of Selection Pressure for Duplicated Gene Pairs

TBtools [58] was used to display the introns and exons of gene structure. The location information of all confirmed cotton SBT, including the starting position, located chromosomes and chromosome length, was obtained from CottonGen [31] (https://www.cottongen.org/, accessed on 15 September 2021) database. The evolutionary tree was constructed by FastTree. For the other cotton species, protein sequence alignment was completed. The cotton protein sequence was BLASTP, and the parameter e-value E-5 and max target seqs was 5. With MCSanX [64] software, use parameters MATCH_SCORE: 50, MATCH_SIZE: 0, GAP_PENALTY: 0, OVERLAP_WINDOW: 0, E_VALUE: 1, MAX GAPS: 25. Collinearity analysis was performed on four cotton species, and the visual collinearity display was completed by TBtools of unlimited synteny visualization. Circos was made TBtools advanced circos. The input file of the circos graph is obtained by modules “GXF gene position & Info. extract” and “Text Transformat for Micro-Synteny View”. Ka/Ks analysis was performed using TBtools’ Simple Ka/Ks calculator.

### 4.4. Co-Expression Network Analysis and Functional Enrichment Analysis

Cotton transcriptomic data and gene co-expression relationship were obtained from ccNET database [32], and *Arabidopsis thaliana* co-expression gene relationship was obtained from ATTED-II [65] (http://atted.jp/, accessed on 8 June 2022) database, with parameters of M.c7.1 and MR30. Amount of gene expression data from paper [66], cytoscape drawing co-express network, the differentially expressed genes were the log2-fold change in FPKM value between treatment and WT (NaCl/CK or PEG/CK) ≤−1 or ≥1. The obtained cotton co-expressed genes were queried by CottonGen [31] (https://www.cottongen.org/, accessed on 15 September 2021) database to obtain *Arabidopsis thaliana* lineal homologous genes, and enrichment analysis and SEACOMPARE analysis were performed on agriGOV2 [40] (http://systemsbiology.cau.edu.cn/agriGOv2/index.php, accessed on 10 June 2022) platform. The most detailed entry in the DAG was selected for SEACOMPARE analysis.

### 4.5. Three-Dimensional Structure Prediction for SBT Protein

Firstly, the protein sequence was BLASTp (https://www.uniprot.org/blast, accessed on 8 January 2023) on the UniProt website to find the protein ID number with the highest score of the corresponding species, where the parameter target database was UniProtKB with 3D structure predictions (alphafold2), and the rest parameters were default. Then enter the obtained protein ID into the alphafold2 website (https://alphafold.ebi.ac.uk, accessed on 8 January 2023) [67,68] and download the PDB file. Finally, the three-dimensional structure of the protein was visualized on pymol.

## 5. Conclusions

In this study, 146 *Gossypium hirsutum*, 138 *Gossypium barbadense*, 89 *Gossypium arboreum* and 84 *Gossypium raimondii* were identified by phylogenetic analysis and domain analysis, and they were divided into six subfamilies. Gene structure analysis of each subfamily showed that most SBT1 gene family members of four cotton varieties did not have introns. The distribution of the SBT gene family members on the chromosomes of the four cotton species was found to be uneven. Through co-expression network analysis, Cotton_A_03171, Cotton_A_03377, Cotton_A_35816, Cotton_A_08556, Cotton_A_18889 and Cotton_A_07447 were found to be in the same network. Most of the genes in the roots, stems and leaves of *Gossypium arboreum* were down-regulated after salt and drought treatment. This network is involved in root tissue development and the response to salt stress in cotton. Co-expressed genes of *Gossypium hirsutum* and *Arabidopsis thaliana* were mostly down-regulated after salt treatment, indicating that the networks were functionally conserved. We predicted that these five *Gossypium arboreum* SBT1 and their corresponding *Gossypium hirsutum* ortholog genes responded to salt stress. This study provides a basis for understanding the biological functions of the SBT family genes in cotton under breeding and abiotic stress conditions. It provides a reference for mining the key genes for breeders to provide candidate genes related to salt and alkali resistance among many genes, and reduce the time taken for breeding varieties.

## Figures and Tables

**Table 1:** Statistics of SBT gene family members of four cotton species.

	*Gossypium hirsutum*				*Gossypium barbadense*				*Gossypium arboreum*	*Gossypium raimondii*
	A Subgenome	D Subgenome	Scaffold	Total	A Subgenome	D Subgenome	Scaffold	Total	A Genome	D Genome
SBT1	25	23	2	50	26	25	0	51	34	27
SBT2	7	7	0	14	7	8	0	15	8	8
SBT3	5	6	0	11	6	6	0	12	6	8
SBT4	14	24	0	38	11	15	1	27	27	23
SBT5	11	9	1	21	13	10	0	23	10	11
SBT6	7	5	0	12	4	6	0	11	4	7
Total	69	74	3	146	67	70	1	138	89	84

## Data Availability

The data and materials that support the findings of this study are available from the corresponding author upon reasonable request.

## Supplementary Materials

The following supporting information can be downloaded at: https://www.mdpi.com/article/10.3390/ijms24065760/s1.
