# Supplementary material for: Genome-Wide Investigation and Co-Expression Network Analysis of SBT Family Gene in Gossypium
Source: Int J Mol Sci. 2023 Mar 17;24(6):5760. doi: 10.3390/ijms24065760 (PMC10056545; doi:10.3390/ijms24065760)
Supplement: Supplementary file 1 [file ijms-24-05760-s001.zip › Supplementary_materials_rev02n.pdf]

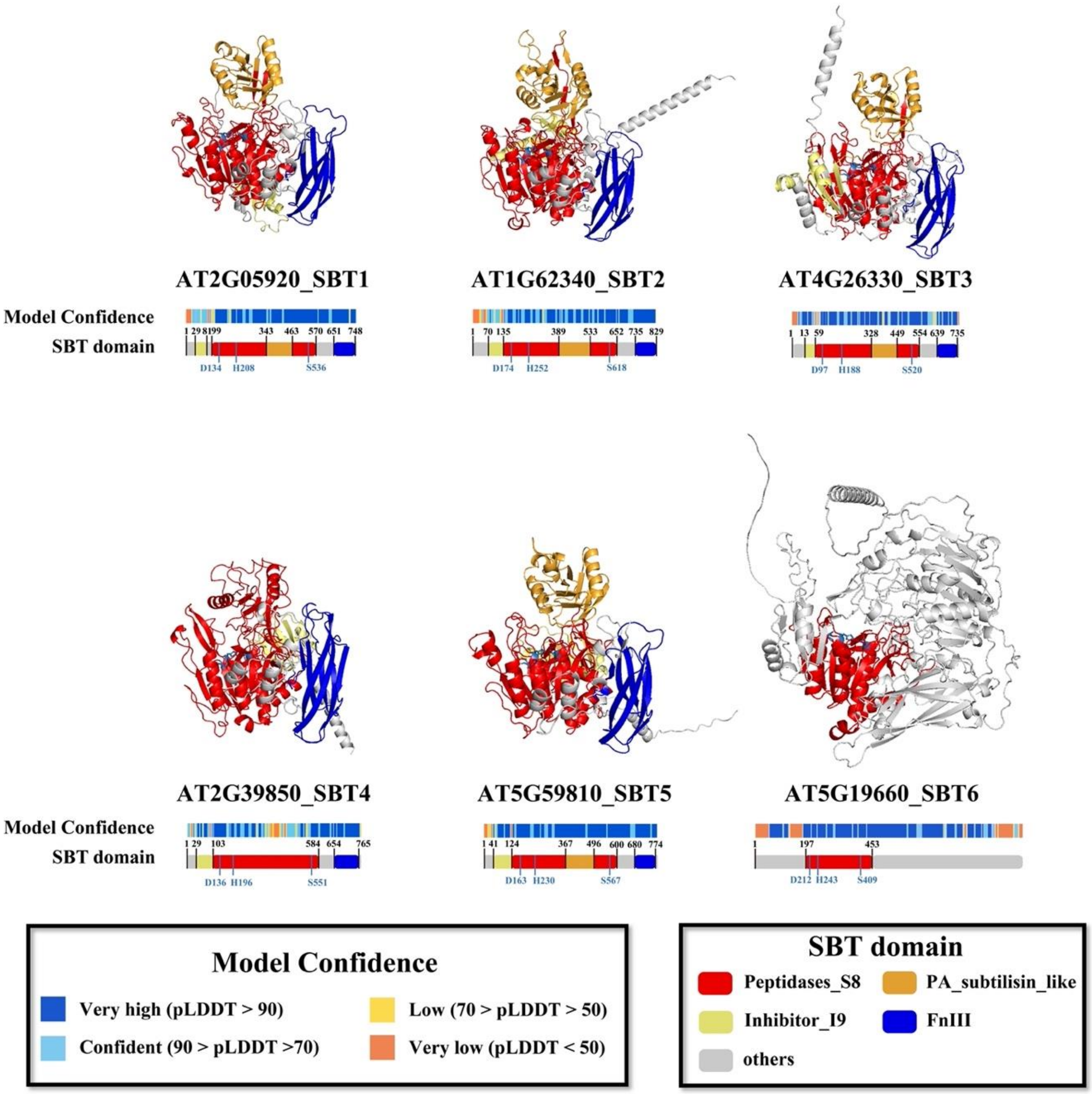

Figure S1. Three-dimensional structure of selected proteins in *Arabidopsis thaliana* SBT1, SBT2, SBT3, SBT4, SBT5, and SBT6. The side chains of the active-site Asp, His, and Ser residues are shown in sky blue. The model reliability was calculated by alphafold2. Blue means high confidence, light blue means high confidence, yellow means low and orange means very low. The SBT domain consists of 5 parts, dark yellow is Inhibitor_I9, dark blue is FnIII, red is Peptidases_S8, brown is PA_subtilisin_like and grey is others.

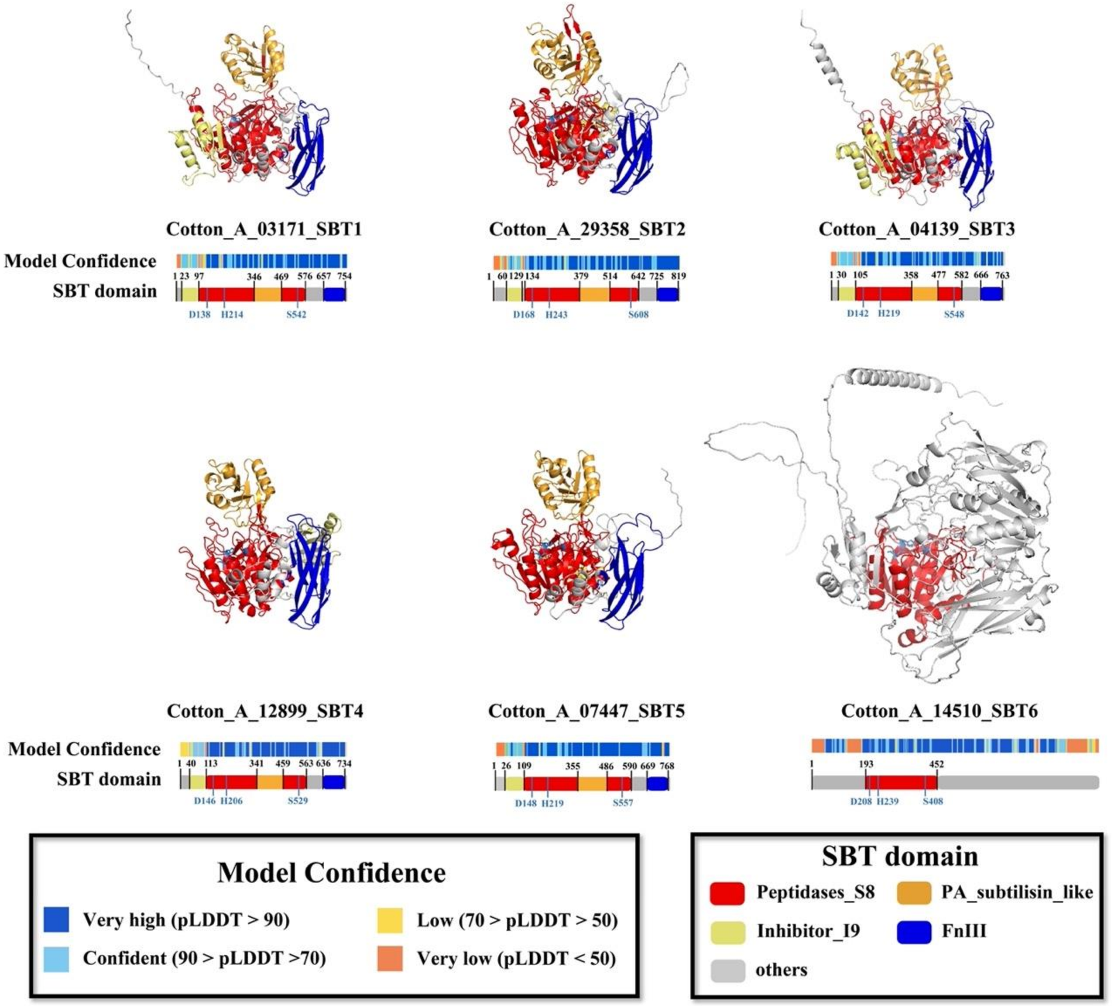

Figure S2. Three-dimensional structure of selected proteins in *Gossypium arboreum* SBT1, SBT2, SBT3, SBT4, SBT5, and SBT6. The side chains of the active-site Asp, His, and Ser residues are shown in sky blue. The model reliability was calculated by alphafold2. Blue means high confidence, light blue means high confidence, yellow means low and red means very low. The SBT domain consists of 5 parts, dark yellow is Inhibitor_I9, dark blue is FnIII, red is Peptidases_S8, brown is PA_subtilisin_like, grey is others.

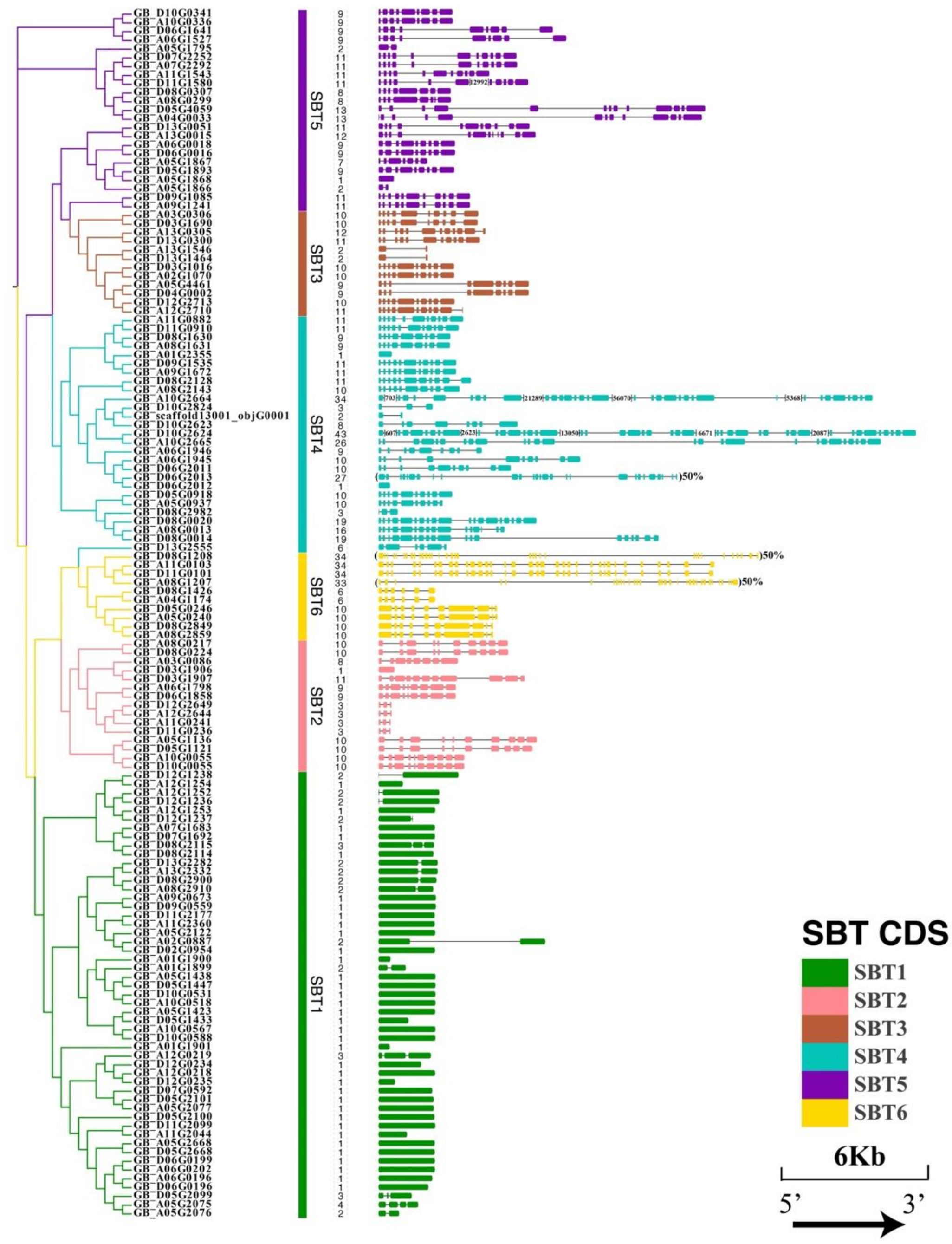

Figure S3. Genetic structure of *Gossypium barbadense*. The squares are CDS, the horizontal lines are introns, and the yellow dots are exons. The evolution tree is built by FastTree. Different colors are used to distinguish the each SBT subfamily. Dark green, pink, brown, cyan, purple, and dark yellow lines represent subfamilies from SBT1 to SBT6, respectively. The dotted line is exon number.

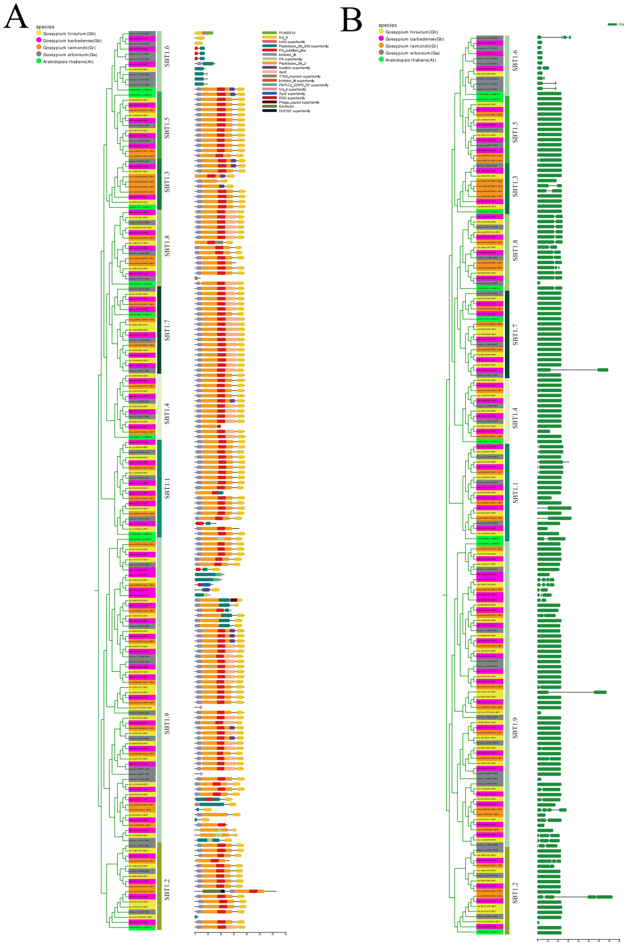

Figure S4. (A) The domain of SBT1 gene family members. Different colors represent different domains. In key domains, red represents PA subtilisin like, yellow represents fn3_6, grey represents Inhibitor I9, orange represents Peptidases_S8_3 (B) The gene structure of SBT1 gene family members. Green represent CDS.

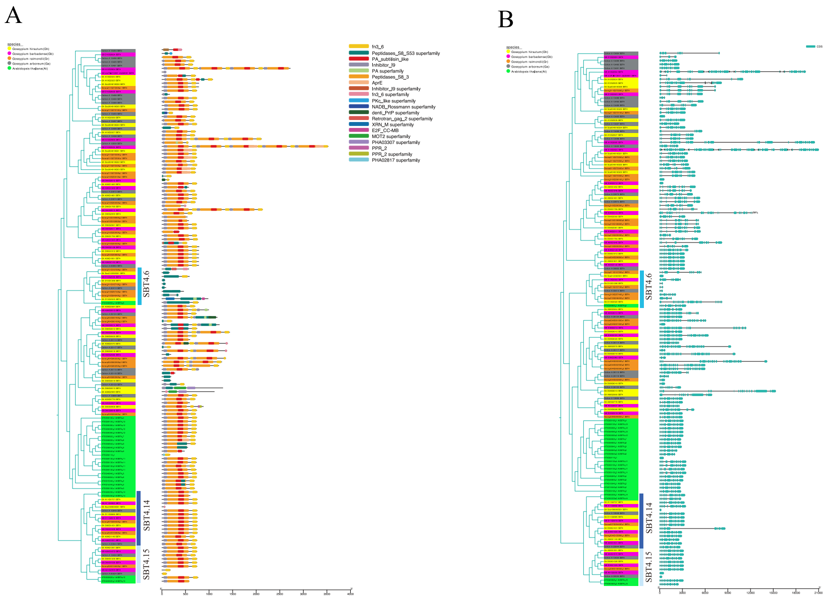

Figure S5. (A) The domain of SBT4 gene family members. Different colors represent different domains. In key domains, red represents PA subtilisin like, yellow represents fn3_6, grey represents Inhibitor I9, orange represents Peptidases_S8_3 (B) The gene structure of SBT4 gene family members. Green represent CDS.

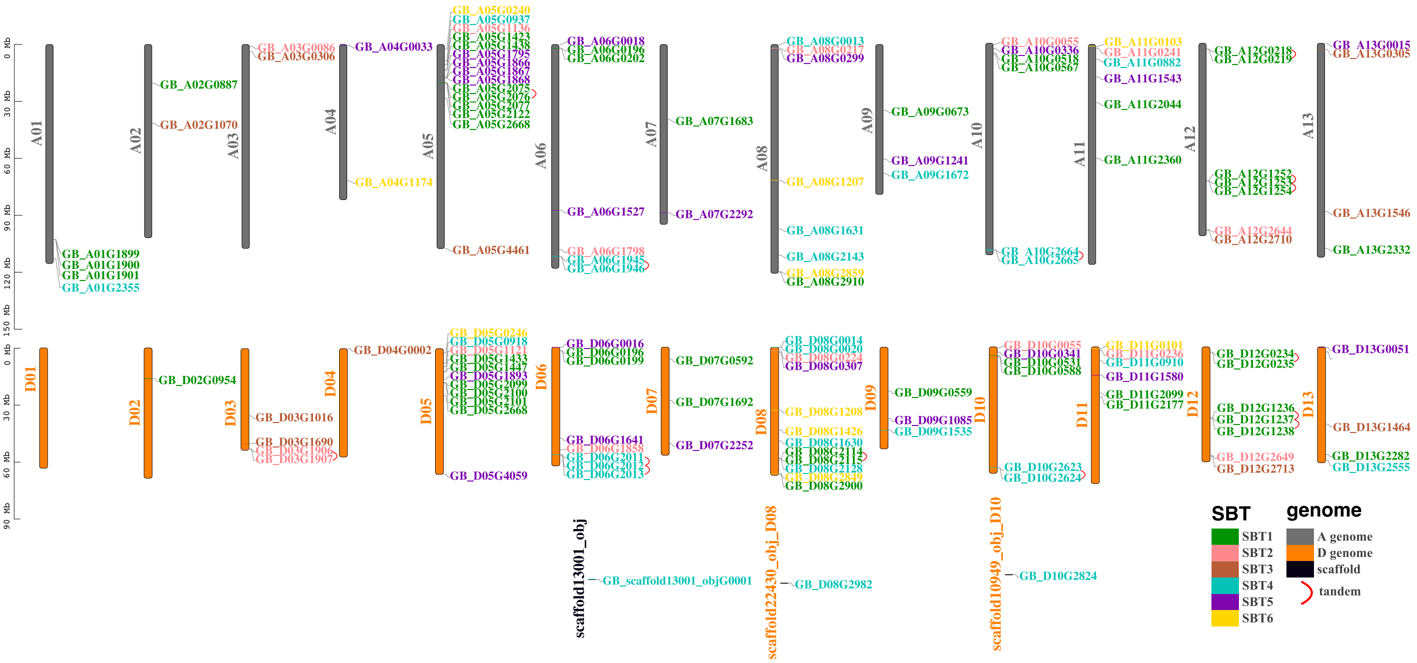

Figure S6. Chromosome positions of SBT gene family members in *Gossypium barbadense*. Location of SBT gene in chromosomes of *Gossypium barbadense*. The grey bars represent the chromosomes of the A subgenome. The orange bar shows the chromosomes of the D subgenome. Black represents unpredicted subgenomic classifications. Different color gene names represent different classifications. Dark green, pink, brown, cyan, purple, and dark yellow lines represent subfamilies from SBT1 to SBT6, respectively. The red curve connects tandem repeats, gene locations and chromosome sizes estimated in Mb on the left side of the figure.

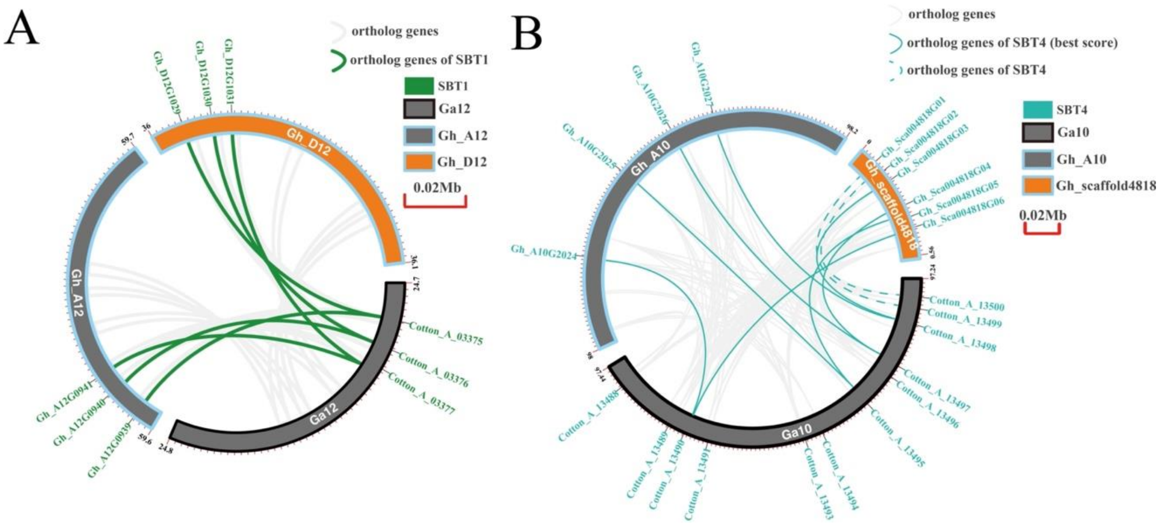

Figure S7. Syntenic relationships among selected SBT genes of *Gossypium arboreum* and *Gossypium hirsutum*. Synteny analysis of selected tandem genes of SBT1 (A) and SBT4 (B). The light blue border of the bar represents *Gossypium hirsutum* chromosome and the black border represents *Gossypium arboreum*. The orange bar shows the chromosomes of the *Gossypium hirsutum* D subgenome. *Gossypium arboreum* and *Gossypium hirsutum* A subgenome were shade with grey. The syntenic of different SBT subfamilies were connected by different colored lines. Dashed lines indicate lineal homologous pairs with non-highest scores.

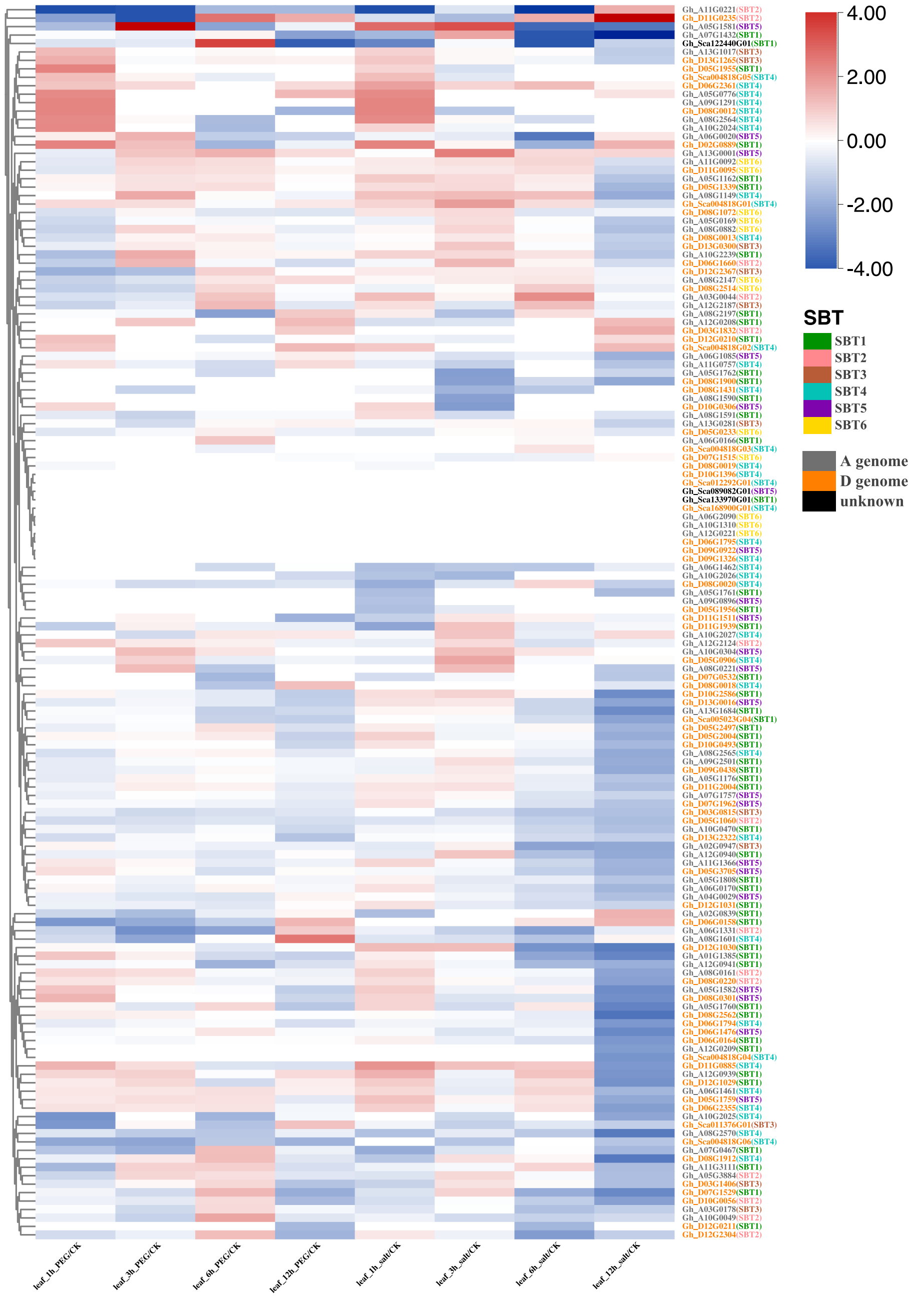

Figure S8. Expression profiles of 146 SBT gene families in *Gossypium hirsutum*. There are 146 *Gossypium hirsutum* gene family members in the vertical, and the ratio of PEG and NaCl treatment to control in leaf in the horizontal sequence, and the classification of each SBT gene family member is at the far right. Different color fonts indicate the each SBT subfamily. Dark green, pink, brown, cyan, purple, and dark yellow lines represent subfamilies from SBT1 to SBT6, respectively. The grey fonts represent the chromosomes of the A subgenome. The orange fonts show the chromosomes of the D subgenome.

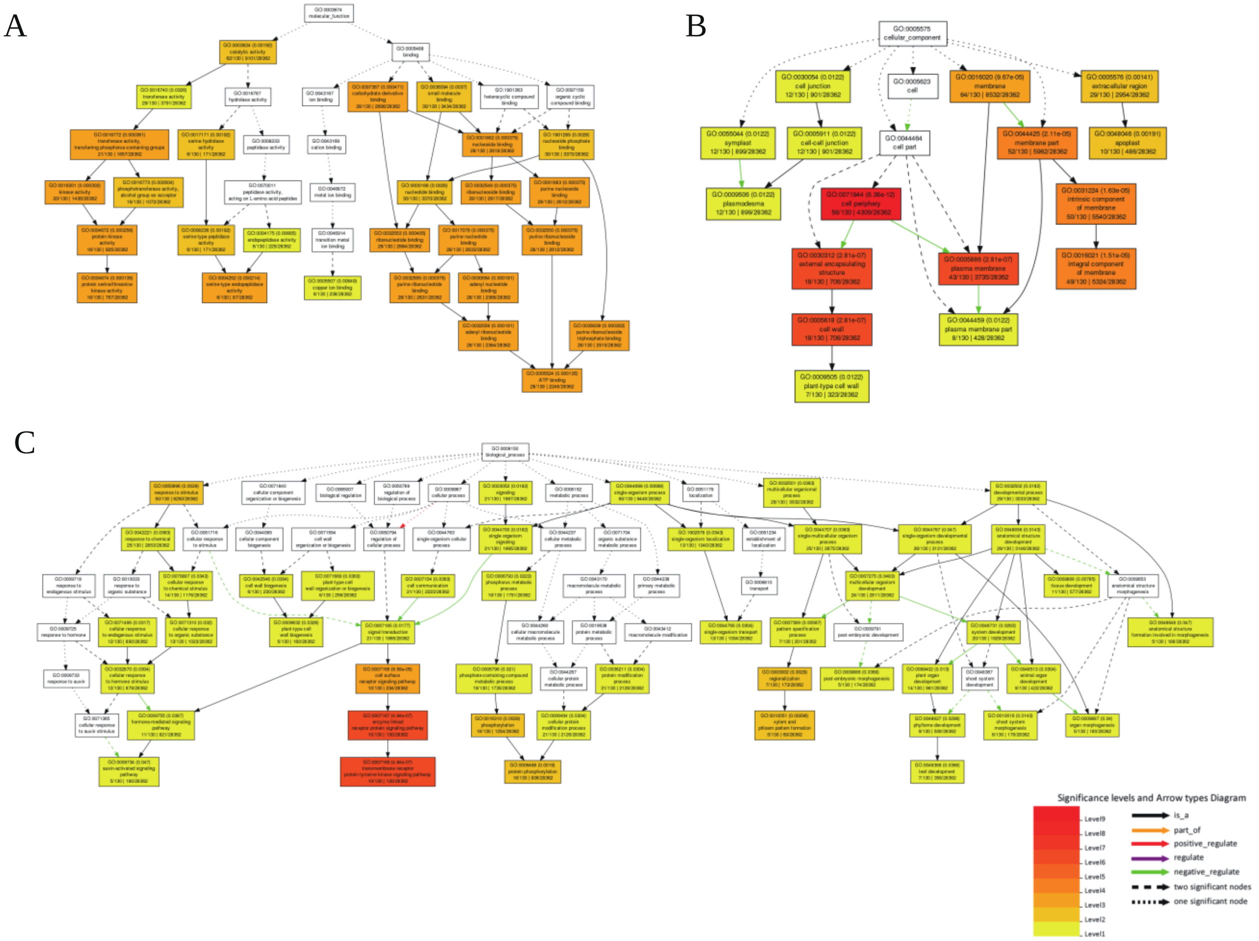

Figure S9. Enrichment results of *Gossypium arboreum* network DAG diagram. (**A**) Molecular Function (**B**) Cellular Component (**C**) Biological Process. The redder the square, the more significant the enrichment of terms.

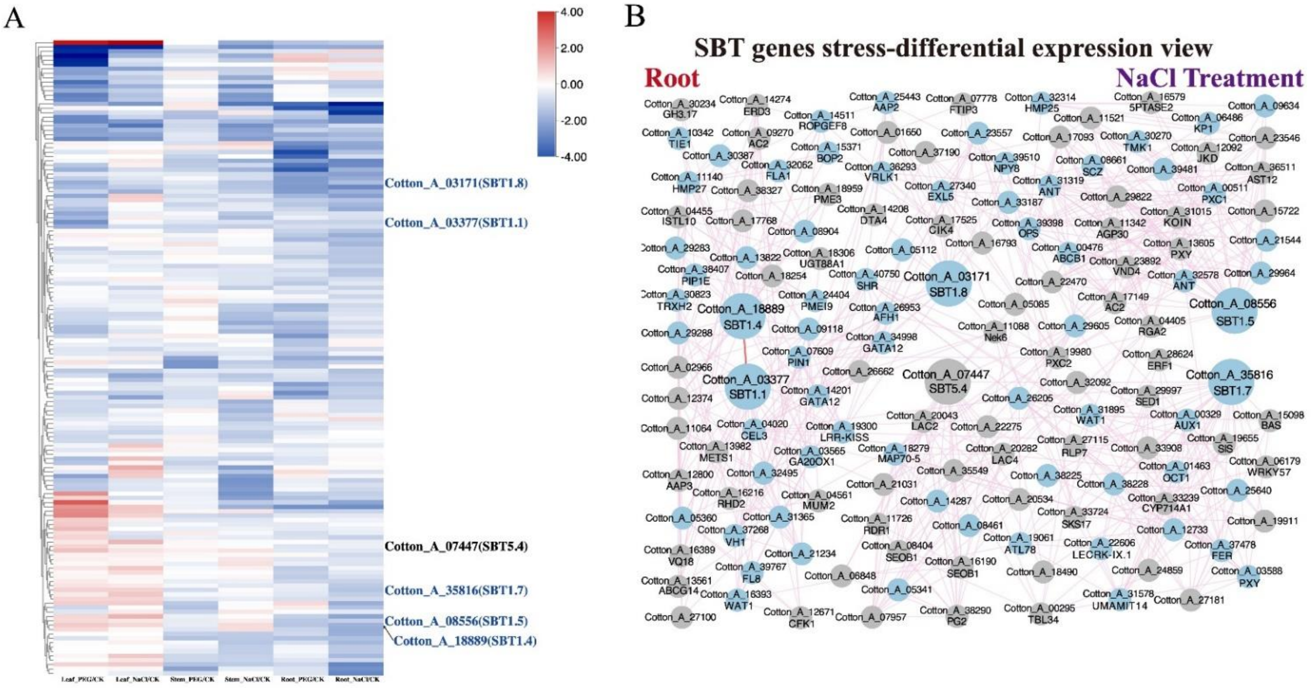

Figure S10. (A) Cluster analysis of gene expression spectrum of six SBT co-expression gene network, longitudinal is 145 co-expression genes, horizontal is, each square represents the expression of each gene under different conditions. Conditions include tissues (leaf, stem, and root) and stresses (PEG and salt). The darker the red is, the higher the expression amount. The gene number on the right side of the heat map represents 6 *Gossypium arboreum* SBT gene family members. (B) Salt treatment co-expression network in root tissue. Blue nodes are down-regulated genes. The pink line shows a co-expression relationship between two genes and the red line indicates SBT gene co-expression. The blue nodes are down-regulated genes and grey nodes are non-regulated genes.

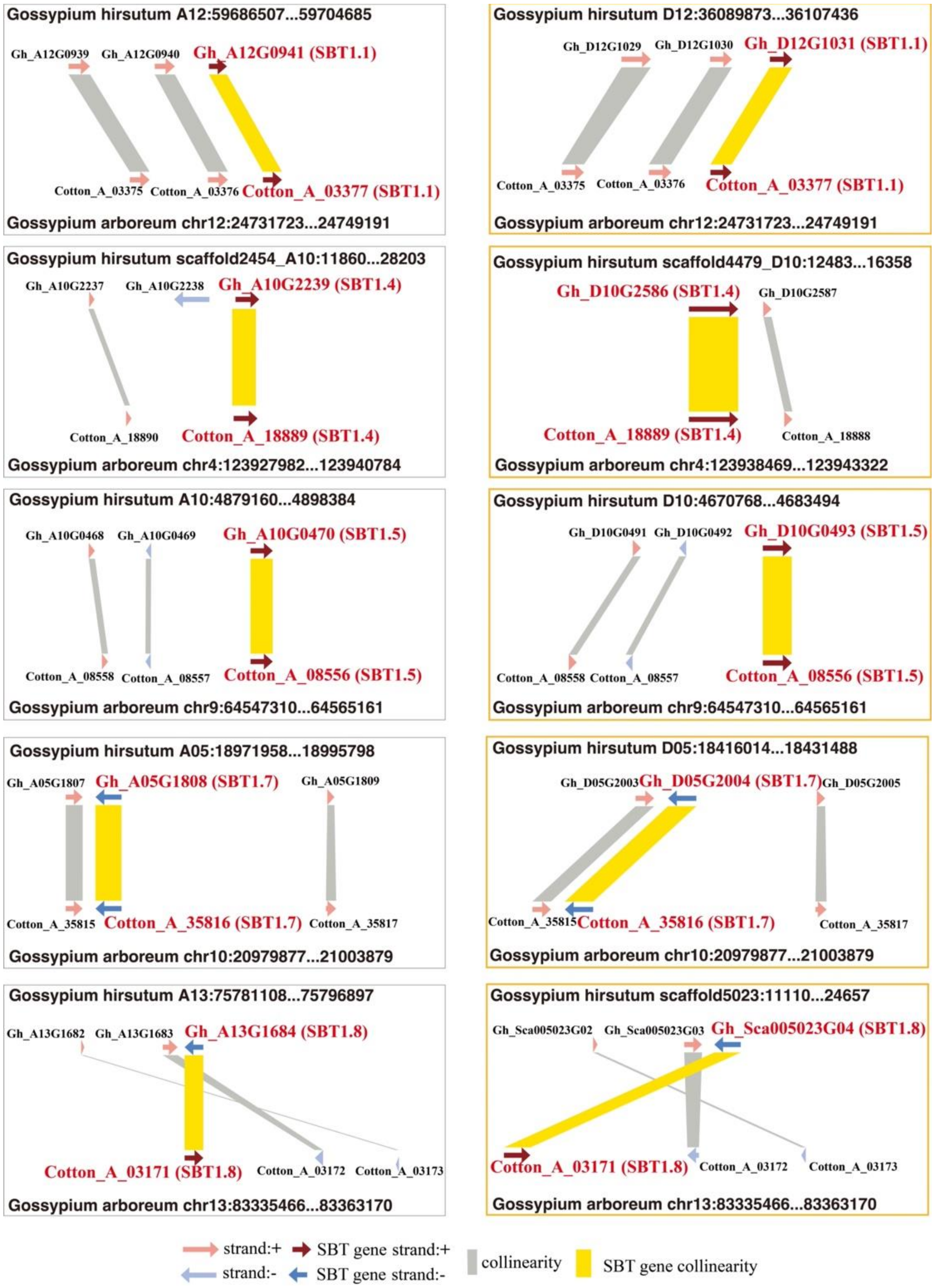

Figure S11. Synteny analysis of five SBT genes in co-expression network between *Gossypium hirsutum* and *Gossypium arboreum*. Gray lines in the background indicate the collinear blocks within *Gossypium hirsutum* and *Gossypium arboreum* genomes, while the yellow lines highlight the syntenic SBT gene pairs. The red letters are members of the SBT gene family. The red arrow to the right indicates sense strands and the blue arrow to the left indicates antisense strands.

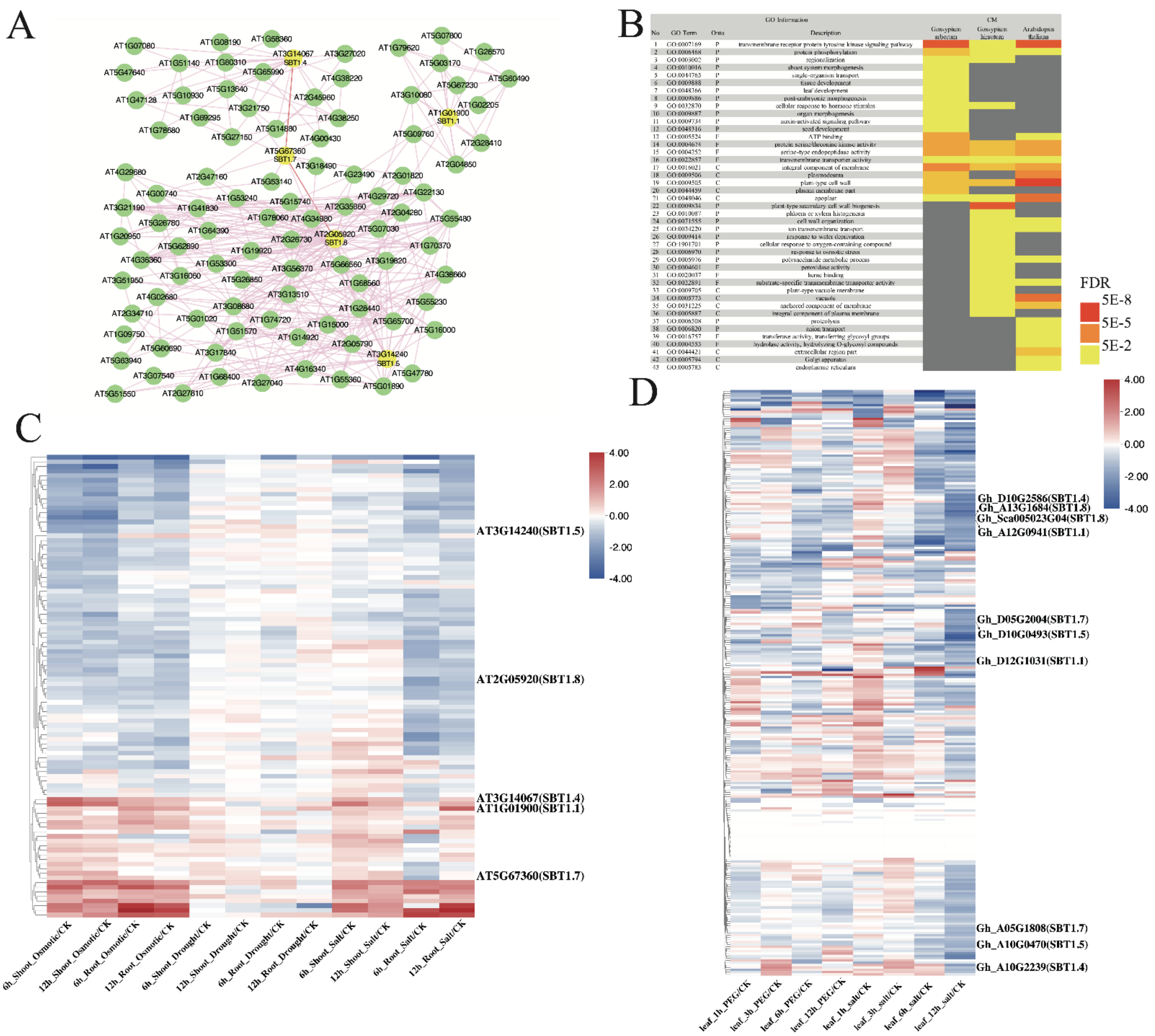

Figure S12. (A) Gene co-expression network of four *Arabidopsis thaliana* SBT gene family members. The green nodes are co-expressed genes, and the yellow nodes are *Arabidopsis thaliana* SBT genes. The pink line shows a co-expression relationship between two genes and the red line indicates SBT gene co-expression. (B) SEACOMPARE analysis for enriched GO terms in genes from *Arabidopsis thaliana*, *Gossypium arboreum* and *Gossypium hirsutum* co-expression network. The redder the square, the more significant the enrichment of terms. (C and D) Cluster analysis of gene expression spectrum of SBT co-expression gene network, longitudinal are co-expression genes, horizontal is, each square represents the expression of each gene under different conditions. Conditions include tissue (shoot and root) and stress (osmotic, drought, and root) in *Arabidopsis thaliana* (C) and conditions include and stresses (PEG and salt) in *Gossypium hirsutum* (D). The darker the red is, the higher the expression amount. The gene number on the right side of the heat map represents SBT gene family members.

Table S1. SBT gene family members of four cotton species.

Table S2. Analysis of gene duplication and evaluation divergence of the SBT family in cotton.

Table S3. Gene expression of *Gossypium arboreum* SBT gene family members

Table S4. Gene expression of *Gossypium hirsutum* SBT gene members.

Table S5. Details of *Gossypium arboreum* co-expression relationship and annotation of co-expression genes

Table S6. Gene expression of *Gossypium arboreum* co-expression network

Table S7. Orthologous gene pairs of SBT proteins in cotton

Table S8. Details of *Gossypium hirsutum* co-expression relationship

Table S9. Details of *Arabidopsis thaliana* co-expression relationship

Table S10. Gene expression of *Arabidopsis thaliana* co-expression network

Table S11. Gene expression of *Gossypium hirsutum* co-expression network
